# Using a Control to Better Understand Phyllosphere Microbiota

**DOI:** 10.1371/journal.pone.0163482

**Published:** 2016-09-26

**Authors:** Andrea R. Ottesen, Sasha Gorham, Elizabeth Reed, Michael J. Newell, Padmini Ramachandran, Travis Canida, Marc Allard, Peter Evans, Eric Brown, James Robert White

**Affiliations:** 1 Molecular Methods and Subtyping Branch, Division of Microbiology, Office of Regulatory Science, Center for Food Safety and Applied Nutrition, FDA, College Park, Maryland, United States of America; 2 Wye Research and Education Center, University of Maryland, Queenstown, Maryland, United States of America; 3 Food Safety and Inspection Service, USDA, Washington D. C., United States of America; Universite Paris-Sud, FRANCE

## Abstract

An important data gap in our understanding of the phyllosphere surrounds the *origin* of the many microbes described as phyllosphere communities. Most sampling in phyllosphere research has focused on the collection of microbiota without the use of a control, so the opportunity to determine which taxa are actually driven by the biology and physiology of plants as opposed to introduced by environmental forces has yet to be fully realized. To address this data gap, we used plastic plants as inanimate controls adjacent to live tomato plants (phyllosphere) in the field with the hope of distinguishing between bacterial microbiota that may be endemic to plants as opposed to introduced by environmental forces. Using 16S rRNA gene amplicons to study bacterial membership at four time points, we found that the vast majority of all species-level operational taxonomic units were shared at all time-points. Very few taxa were unique to phyllosphere samples. A higher taxonomic diversity was consistently observed in the control samples. The high level of shared taxonomy suggests that environmental forces likely play a very important role in the introduction of microbes to plant surfaces. The observation that very few taxa were unique to the plants compared to the number that were unique to controls was surprising and further suggests that a subset of environmentally introduced taxa thrive on plants. This finding has important implications for improving our approach to the description of core phytobiomes as well as potentially helping us better understand how foodborne pathogens may become associated with plant surfaces.

## Introduction

Culture independent phyllosphere research has greatly expanded our understanding of the diversity of microbes associated with plant surfaces [1–13]. Food safety initiatives have played a small but important role in the advancement of culture independent phytobiome research. The microbiology of living surfaces of fresh produce has clear implications for public health and food safety [14]. The description of agro-ecologies (beginning with crop phytobiomes) along the farm to fork continuum has begun to establish microbial baselines that will contribute to an improved understanding of precisely where and how human pathogens may become associated with food plant ecologies in agricultural settings. At least nineteen *Salmonella–*tomato associated outbreaks occurred between the years of 1990 and 2014, causing thousands of illnesses (FDA internal document) [7]. Understanding how contamination events occur is extremely important and thus, the tomato microbiome has become an important study system for food safety research.

Agricultural management practices for food crops have been studied to better understand the role they may play in shaping phyllosphere microbiota. For example, the impact on phyllosphere microbiota by different irrigation waters has been studied [15, 16] as well as the impact of different pesticide schedules [17–19] and even organic and conventional management [8]. Seasonality and biogeography have also been contrasted to farming systems[20–22]. An interesting trend that was observed in many of these studies was the lack of statistically significant differences in plant microbial communities that correlated to the treatment queried. For example, Telias et al. studied water sources used in agriculture (ground water compared to surface pond water) and showed that the microbial communities of water sources were highly divergent, but the communities collected from tomatoes treated with the different waters did not exhibit those same differences [15, 16] This suggests that environmental pressures (potentially air) in the phyllosphere exerted stronger pressures than either water source did. Work by Perazzolli et al. supported this observation by demonstrating that different pesticides used on the same crop had less influence on phyllosphere crop microbiota than biogeographical factors did [22]. They found that epiphytic microbes associated with grape vines were not significantly altered by different pesticides treatments (bio-control and traditional pesticide). Instead, the primary driver of microbial differences appeared to be biogeography–which suggests that environmental parameters such as wind and air associated with each region likely influenced the consortia of microbes found on the grape plant surfaces.

Furthermore, work that examined the influence of two very different pesticides on tomato crop microflora, found that the most striking differences in microbial composition were associated with the sampling time-points and not with the pesticide treatments [18] suggesting once again, that environmental parameters, largely unaccounted for to date, may be the most influential drivers of the microbiology of the phyllosphere. Marine et al. demonstrated that for leafy greens grown in the mid-Atlantic region, seasonal events and weather conditions, as opposed to farming systems, were the most important risk determinants for crop contamination by human pathogens [20]. The overall importance of seasonality on phyllosphere community structure and membership has been demonstrated by both culture dependent and culture independent research studies [23–26].

While these studies all suggest that environmental factors may play the most significant roles in microbiologically seeding the phyllosphere, there is also evidence that host plant species play significant roles in the shaping of phyllosphere microbial ecology and succession[27–30].

While there are undoubtedly important drivers from both host plants and environmental factors, the fact remains that almost every phyllosphere study in the literature to date has sampled microbes from surfaces of plants and described these consortia as phyllosphere microbiota *without any type of control*–such as an inanimate surface placed at a similar elevation to the plant part sampled. Thus we have an opportunity to learn more about drivers of phyllosphere microbiota by employing an inanimate control to help us better understand differences between environmentally introduced microbial species and endemic or host plant mediated microbiota.

To attempt to distinguish between host plant mediated and environmentally introduced microbiota, we interspersed sterilized plastic plants in a row of live tomato plants and sampled each surface type at four time-points throughout a growing season. DNA was extracted and used with 16S rRNA amplicon sequencing to describe bacterial membership for each sample type.

## Results

### Shared and Unique Bacterial Communities

**Fig 1:**
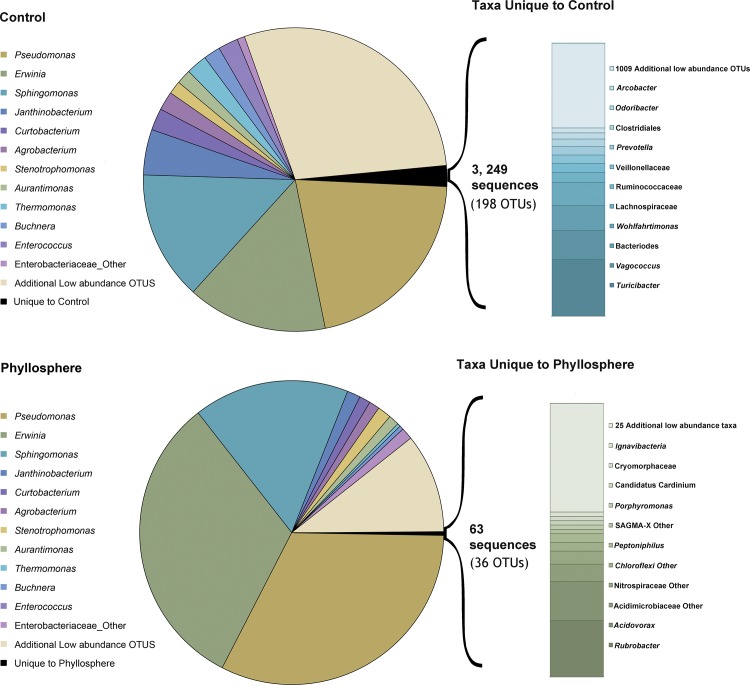
Bacterial Genera Unique to Phyllosphere and Control Samples. Using the total depth of sequences generated for Time-point 0 (16,074 sequences per independent replicate) (N = 9) for phyllosphere (live tomato plants) and control (plastic plants), we were able to identify 63 OTUs that were unique to the phyllosphere environment and 3, 249 OTUs that were unique to the control (plastic plant) environment.

**Fig 2:**
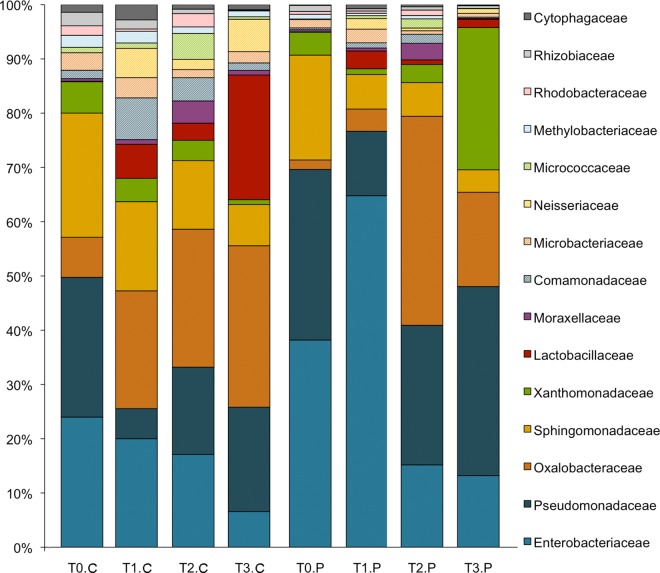
Bacterial Families in Control and Phyllosphere. Percentage of library represented by the most abundant bacterial families identified using 16S rRNA gene amplicons for merged independent replicates of control (C) and phyllosphere (P) at all time-points. Taxonomy was assigned using the RDP classifier trained on the GreenGenes database.

A total of eight independent replicates for each treatment (n = 2) and time-point (n = 4) were used to compare the bacterial composition associated with each surface type (i.e. control (plastic) and phyllosphere). 16S rRNA amplicon sequences were filtered for quality and clustered into operational taxonomic units (OTUs) using the QIIME package (see methods). To normalize for differences in sequencing depth, all replicates were subsampled to 2,500 sequences prior to downstream statistical comparisons. Excluding low abundance taxa–(less than 0.5% of libraries), we observed very high percentages of shared OTUs. Shared bacterial taxa ranged from 92.59% to 100% for all time-points (Table 1). Only 2 unique OTUs were observed in control samples on August 15^th^ 2013, and 2 OTUs unique to phyllosphere were observed on June 30^th^ 2014 and July 31^st^ 2014 (Table 1). When Time-point 0 was examined independently at a deeper level of sequencing depth (16,074 sequences per sample), the percentage of shared taxa remained high at 94.7%. With the inclusion of low abundance OTUs (all those that occurred in less than 0.5% of the data), an interesting trend was observed. For almost every time point–there was a greater diversity of low abundance OTUs present in control samples when compared to phyllosphere samples (Table 2). This implies that the air is host to a greater diversity of microbes than can be found on the surfaces of plants and that a subset of bacterial members thrive in the phyllosphere. This trend was even more pronounced in Time-point 0, when it was examined independently. Without rarefying to 2500 but instead maintaining all 16,074 sequences recovered for each replicate *and* including the low abundance OTUs—3,249 sequences were uniquely associated with controls (representing 198 OTUs) while only 63 sequences (representing 36 OTUs) were uniquely associated with phyllosphere samples (live tomato plants) (Fig 1). The most abundant phyllosphere unique taxa were *Rubrobacter*, *Acidovorax*, *Peptoniphilus*, *Porphyromonas* and undefined members of Acidimicrobiaceae, Nitrospiraceae families and the phylum Chloroflexi. Dominant unique taxa for controls were *Turicibacter*, *Vagococcus*, *Bacteriodes*, *Wohlfahrtimonas*, *Prevotella*, and undetermined members of Lachnospiraceae, Ruminococcaceae and Veillonellaceae families (Fig 1). Profiles of dominant bacterial families observed in our rarefied datasets for all time-points and treatments, including the store microbiota are shown by independent replicate in S1 Fig. Fig 2 shows the most abundant families that were observed in 16S rRNA gene libraries for merged independent replicates of phyllosphere (P) and control (C) samples. Despite the dominance of Enterobacteriaceae in phyllosphere and control samples, high-resolution taxonomic analysis using the Resphera Insight protocol described *Pantoea*, *Erwinia* and *Serratia* species, but found no evidence of *Salmonella* across the sample set in either phyllosphere or control samples.

**Table 1:** Shared and Unique OTUs for Phyllosphere and Control Environments.

Date	Shared OTUs	Unique Control	Unique Phyllo	Percent Shared
**AUG 15**^**th**^ **2013**	**25**	**2**	**0**	**92.59**
**JUNE 30**^**th**^ **2014**	**32**	**0**	**2**	**94.12**
**JULY 16**^**TH**^**2014**	**25**	**0**	**0**	**100**
**JULY 31**^**ST**^**2014**	**22**	**0**	**2**	**91.67**

Using the rarified data set of 2500 sequences per independent replicate, and excluding all sequences that represented less that .5% of the libraries, only 2 OTUs were unique to control samples on August 15^th^ 2013 and 2 were unique to phyllosphere samples at each of two time-points (June 30^th^ and July 31^st^, 2014).

**Table 2:** Unique OTUs in Control and Phyllosphere Environments.

Date	Unique Control	Unique Phyllo
**AUG 15th 2013**	**227**	**33**
**JUNE 30th 2014**	**254**	**72**
**JULY 16TH2014**	**194**	**97**
**JULY 31ST2014**	**285**	**29**

Using the rarified dataset without the .5% cutoff, OTUs unique to control samples were more numerous than OTUs for phyllosphere samples at every time-point.

**Fig 3:**
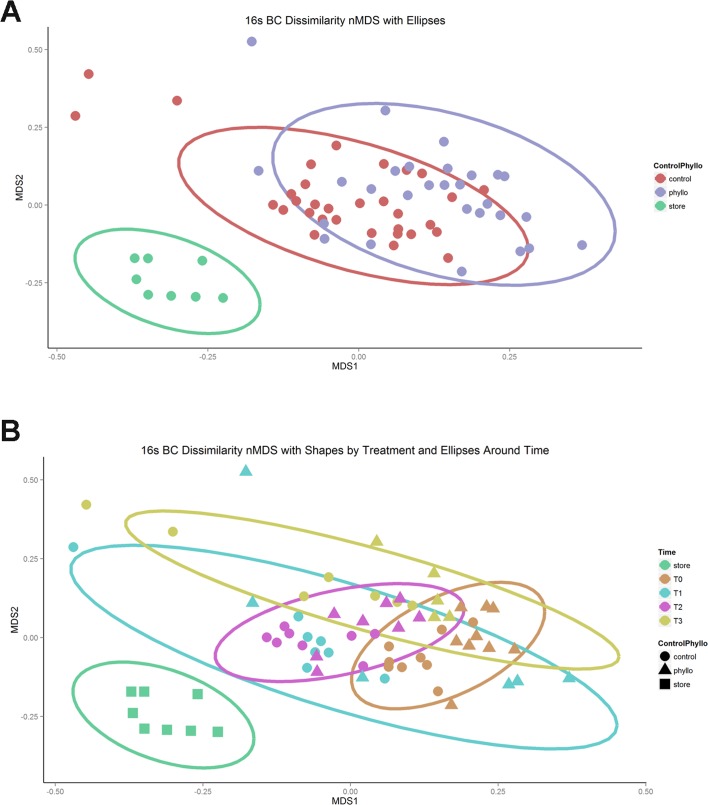
NDMS of 16S Communities from Store, Control and Phyllosphere Environments at 4 time-points. **3a** Bray-Curtis ordination colored by environment (control (plastic), phyllosphere, and store bacterial communities). A very clear separation of store microbiota (green) is evident, however no significant separation between communities associated with control (red) and phyllosphere (purple) is evident. **3b** Bray Curtis ordination colored by time-point. Time-point 0 (T0) and Time-point 1 (T1) appear to separate from each other and also from time-points 2 (T2) and 3 (T3), however T2 and T3 are more similar. For both 1a and 1b, the ellipse defines the upper 95^th^ percentile limit of the assumed distribution.

**Fig 4:**
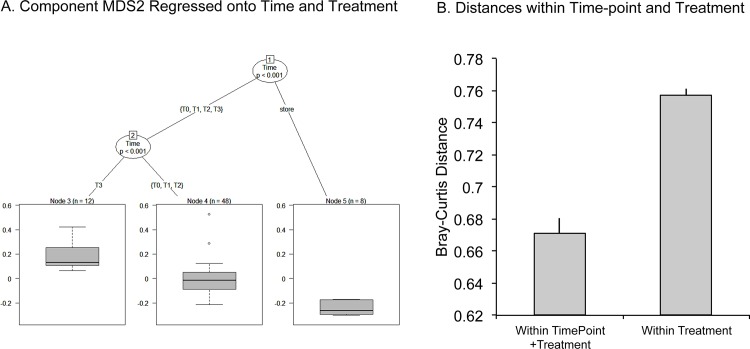
MDS2 Regressed onto Time and Treatment and Distances Within and Between Time and Treatment. **4a** To test the global null hypothesis of independence for covariate variables (store, phyllo, enviro, and time-points: T0, T1, T2, T3) and associated response variables (MDS1 and 2), a conditional inference regression tree was modeled onto the data after nMDS scaling. For ordination component MDS2, the time at which the samples were collected determines their position in the ordination. **4b** Distances between control and phyllosphere samples from the same time-point were significantly closer to each other than they were to treatment (environment) (P = 2e-16; Mann-Whitney).

Little separation by treatment (control and phyllosphere) was evident using nonmetric Multi Dimensional Scaling (nMDS) ordinations to look at Bray Curtis dissimilarity of bacterial communities (Fig 3). Microbiota that was washed off plastic plants pre-surface sterilization however, was clearly different from microbiota associated with plastic and live plants from the field environment. The washing step was performed to ensure that microbiota from the store environment where plants were purchased was not erroneously described as part of the environmentally driven consortia. Fig 3A shows nMDS ordination of 16S libraries for all environments: store, control (plastic plants) and phyllo (phyllosphere, live tomato plants). Fig 3B shows an nMDS ordination of the same data separated by time point. To further test the global null hypothesis of independence for covariate variables (store, phyllo, enviro, and time-points: T0, T1, T2, T3) and associated response variables, a conditional inference regression tree was modeled onto the data. Nodes were generated by performing a binary split on all covariates (where the null hypothesis could not be rejected) using a p value set at 0.1 as a cutoff (Fig 4) [31]. For ordination component MDS2, the time at which the samples were collected determines their position in the ordination (Fig 4A). However, for component MDS1, whether or not the plant was real (phyllo) or plastic (control) does not play a significant role in its location on the ordination chart. Nonparametric significance tests comparing the MDS1 component for time point 0 (p < 0.001) and time-points 1, 2, and 3 (p < 0.001) support this conclusion. The distances between control and phyllosphere samples from the same time-point were significantly closer to each other than they were to other time-points of same treatment (P = 2e-16; Mann-Whitney), suggesting that temporal changes are more influential drivers of community composition than treatment alone (Fig 4B). Additionally, we performed an analysis of multivariate homogeneity of group dispersions (using the permutest in the Vegan package). Significant differences in dispersion were identified between T0 compared to T1 and T1 compared to T2 (P<0.007 for each comparison). We did not identify significant differences in dispersions among time-points within each environment (control and phyllosphere), most likely due to limited group sizes (S2 Fig).

### Differential Abundance of Bacterial Communities

For every time-point there were numerous taxa that were significantly differentially enriched from one treatment to the other. For example, in Time-point 0, five different genus level OTUs in the Family Sphingomonadaceae were differentially enriched–with each one occurring in greater abundance in control samples. There is research that describes the protective effect that specific Sphingomonadaceae taxa can have on host plants [32]. There is even a species of *Sphingomonas* with the species epitaph, “*phyllosphaerae”* [33]. The enhanced abundance of *Sphingomonas* OTUs in control samples suggests that these taxa may not actually be endemic to the phyllosphere but rather, adapted to this niche.

**Fig 5:**
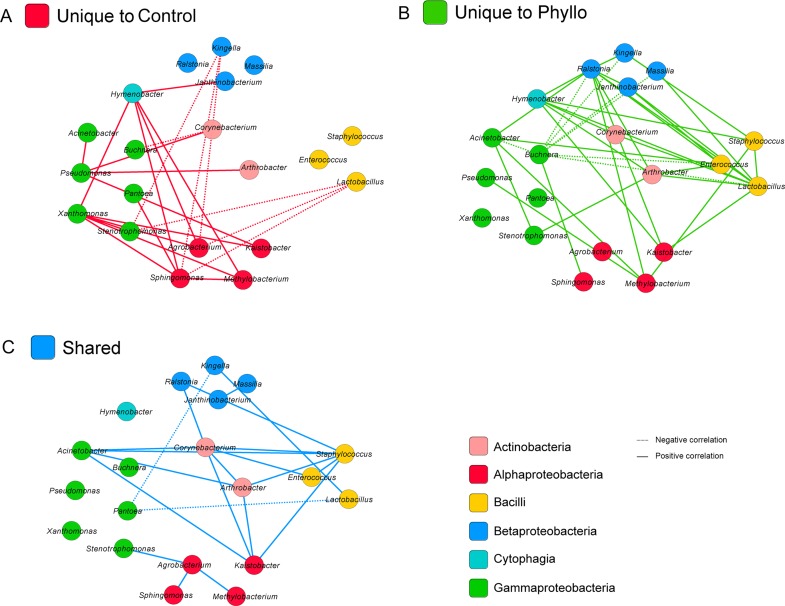
Network relationships in Control and Phyllosphere. Computing Spearman’s correlation coefficients with associated P values, (P< 0.05) after correction using FDR for all pairwise relationships of genera in control and phyllosphere samples (using Cytoscape v3x for visualization www.Cytoscape.org), 21 pairwise correlations were shared (C) between control and phyllosphere samples. A total of 37 correlations were unique to phyllosphere (B) and 23 correlations were unique to controls (A). Correlations unique to the phyllosphere appear increased among members of Bacilli, and Betaproteobacteria, including genera such as *Ralstonia*, *Staphylococcus*, and *Arthrobacter*. For example, *Ralstonia* has many significant relationships in phyllosphere samples but none in controls.

The composition itself (shared incidence of each taxa in each sample) was highly similar for both sample types. The differential abundance of specific groups described above provided the first insight into microbiota that were responding to host plant physiology in contrast to environmental deposition. To further explore bacterial relationships thriving in response to biological and physiological drivers from living leaves, we performed a network analysis by computing Spearman’s correlation coefficients with corresponding P values for all pairwise distances of bacteria in phyllosphere and control samples (Fig 5). Examining significant pairwise correlations (P< 0.05 with False Discovery Rate (FDR) correction[34], a total of 23 unique correlations were identified in control samples. For phyllosphere samples, 37 unique correlations were observed and 21 significant correlations were shared between the two sample types (Fig 5).

## Discussion

Work supporting the observation that microbial consortia associated with plant surfaces may be heavily influenced by air was recently presented at the 15^th^’International Symposium for Microbial Ecology (2014) [35]. Looking to understand the origins of the microbes that inhabit clouds, Santl-Temkiv et al. examined 16S rRNA and rDNA from soil, water, plant surfaces and air, and showed that communities from air and plant surfaces were the most similar [35]. Work by Gales et al., actually demonstrated that plant surfaces are useful for the monitoring of bioaerosol emissions from a composting plant [36] because of their accurate representation of how far airborne microbiota actually travel from the composting plant. As previously mentioned, a number of studies suggest that seasonal and/or biogeographic factors (potentially airborne or other environmental pressures) appear to have a stronger influence on the composition of phyllosphere microbiota than pesticides [18], water sources [15] and other agricultural management practices [20, 22, 25].

In contrast to these findings, other work has described phyllosphere and air bacterial communities as distinct from one another other, with only 18% of OTUS at 97% shared in a study done comparing greenhouse air (represented by glass slides placed adjacent to plants) and *Arabidopsis thaliana* plant surfaces [30, 37]. Maignien et al. describe the combination of selective and random forces that shape the microbial ecology of epiphytic bacterial populations of surfaces of *Arabidopsis thaliana* leaves grown in a green house. They suggest that proximity of plants may play a role in the bacterial community development and succession but they also acknowledge that random and stochastic processes appear to contribute to the development of phyllosphere bacterial communities that remain distinct from greenhouse air communities. The greenhouse air however, was constant in temperature and without wind pressures and thus does represent the pressures that occur in field conditions. Indeed, the authors mention that greenhouse *Arabidopsis* phyllosphere communities differed from those of field grown plants. Research by Williams et al. also supports the observation that microbiota of laboratory grown plants is significantly distinct from that of plants grown in field conditions [38]. Interestingly however, and consistent with the results presented here, Maignien et al. described a greater diversity of unique OTUs (1,003) associated with greenhouse air (measured using deposition on glass slides) compared to surfaces of greenhouse grown plants (435 OTUs). This observation supports the hypothesis that microbial colonizers of the phyllosphere represent a subset of the microbiota associated with air. A second study that suggests that microbiota from plant surfaces is distinct from air microbiota was conducted by Vokou et al. [37]. However, the total number of sequences generated for this study was not sufficient to make robust inference about the scope of alpha or beta community diversity for phyllosphere or air communities.

While air may play a poorly described but important role in phyllopshere microbial composition, it is well documented that host plants themselves mediate important microbial dynamics in the phyllosphere. We saw evidence of this in the differential abundance of certain taxa between control and phyllosphere samples. Especially within the family Sphingomonadaceae. Other important evidence of influence of host plant on phyllosphere bacterial microbiota was evident in the network analyses (Fig 5). As previously mentioned, 21 correlations were shared between the two sample types with 37 unique to phyllosphere in contrast to only 23 unique to controls. A greater number of unique phyllosphere relationships, despite the fact that there were less unique phyllosphere OTUs, demonstrates the robust influence of host plant biology on phyllosphere microbiota. Correlations unique to the phyllosphere appeared among members of Bacilli, and Betaproteobacteria including genera such as *Ralstonia*, *Staphylococcus*, and *Arthrobacter* (Fig 5). The examination of the *Ralstonia* node for control and phyllosphere samples is particularly striking. *Ralstonia* is an important and well known plant pathogen of many plants. *Ralstonia solanacearum* causes wilt in a variety of plants–notably tomato. A complex network of relationships between *Ralstonia* and other bacteria is easily observed in phyllosphere while no relationships between *Ralstonia* and other genera are evident in control samples (Fig 5).

## Conclusions

The most surprising observation for the bacterial taxa associated with control and phyllosphere samples was the high percentage of shared taxonomy for each sample type. These findings suggest that airborne or other environmental pressures may play an important role in driving the consortia of microbes that are associated with phytobiomes. These results also highlight the need for enhanced sampling methodologies to more comprehensively describe endophytic and epiphytic core phytobiomes. More sophisticated air sampling will be needed for future work to exclude the possibility that live plants may have introduced bacterial microbiota to controls. A repertoire of “usual suspects” spanning numerous bacterial and fungal phyla has been reported across a variety of plant phytobiomes, however the delineation of a core microbiome for any plant remains elusive. Efforts to describe core endophytes are closer to accomplishing this goal than efforts to describe core epiphytes. Indeed, the delineation of a “core microbiome” for the well studied human GI tract also has yet to be established. There are so many genetic, temporal, physiological, and stochastic environmental factors that exert small yet significant pressures on undescribed and interwoven ecologies.

A research goal for both human microbiome and phytobiome research is the description of core microbiota and how endemic, established or introduced taxa play a role in community stability, persistence, and antagonism or suppression of pathogens that enter the host microbiome. The observations presented here using plastic plants as a control surface, and by Maignien et al., using glass slides, demonstrated that more bacterial diversity was associated with representative air samples (plastic plants and glass slides) than with surfaces of plants. This suggests that improved methods need to be developed in replicated and diverse biogeographic regions before a core phytobiome can be described for any plant species. The great diversity of microbes in agricultural air systems is worthy of further study with state of the art air monitoring and integrative data techniques. Because of the proximity of the plastic plants used in this study to the live plants, we cannot rule out the possibility that the community composition of plastic plants actually represents local dispersal from colonizers of living tomato plants. However this does not explain the augmented diversity of OTUs associated with the control environment. The many (37) significant unique correlations between genera in phyllosphere samples observed in the network analysis (Fig 5) definitely provide an exciting insight into relationships that are host plant mediated. There was also evidence of many shared relationships between the two sample types as well (21) (Fig 5). These results suggest that stochastic processes, including wind driven dispersal and drift may play a significant role in the structuring of phyllosphere communities. They also provide very interesting insight into some of the potentially host plant mediated dynamics of the phytobiome. Continued examination of both control and phyllosphere samples will help us improve our understanding of which microbiota in phyllosphere communities are host plant mediated and which may be introduced by environmental forces.

## Materials and Methods

### Field Collection

Commercial tomato plants, variety BHN 602, were planted a minimum of 60 cm (2 feet) apart in 23 meter rows comprised of approximately 25 plants at the Wye Research and Education Center of the University of Maryland. The plants were border plants surrounding another experiment and received no treatment inputs such as pesticide applications. Plastic plants (figs) were purchased from Ikea to serve as non living controls possessing roughly same color, height and surface area of mid season tomato plants. The selection of the plastic plants for use as a control was made based on similarity of height, color, and surface area of plastic leaves to live tomato leaves. Several other materials were used in preliminary trials such as yellow and green sticky cards but these were determined to be too differentially selective for insect microbiota and DNA recovery was also significantly more challenging. The lack of sticky surface associated with the plastic plants provided a less biased surface to estimate bacterial deposition by environmental forces. Cost and availability were also practical considerations for selections of the controls. Plastic plants were washed thoroughly and rinsed with a 3% bleach solution for “sterilization”. Plants were approximately 60 centimeters tall, which matched the height of the tomatoes at sampling time. Plastic plants were interspersed between live plants with a range of approximately 30 centimeters to 60 cm at the base of each plastic and live plant. All plants were supported by the same twine system, installed to support the tomato plants. In general, plastic and live plants were spatially separated by at least 12 cm at leaf tips, however this distance became smaller for a few live and plastic plant pairs over the six weeks of the experiment. In 2013 tomato phyllosphere samples and plastic leaves were collected on August 15, 2013 after plastic plants had been in the field for approximately three weeks. In 2014, plastic plants were placed in the field on June 13^th^, and sampled alongside real tomato leaves at three time points at two week intervals, June 30th, 2014, July 16^th^, 2014 and July 31^st^, 2014. Thus, at the first collection, samples had been in the field for two weeks, at second collection samples had been in the field for 4 weeks and by the third collection samples had been in the field for 6 weeks. Time-points are described as Time-point 0 (T0: August 15^th^ 2013), Time-point 1 (T1: June 30^th^, 2014, Time-point 2 (T2: July16^th^, 2014) and Time-point 3 (T3: July 31^st^ 2014). Additional sequencing of the microbiota associated with the plastic plants post purchase and pre- washing and “sterilization” was performed to provide an understanding of how effective our washing techniques were and to ensure that diversity described from the controls did not represent the store environment. Using sterile water and sonication, store microflora wash was removed, centrifuged and DNA was extracted from the pellet. All plastic and living leaves were collected from similar altitudes on the adjacent plants and from the front of each plant to ensure that no plastic and living leaves that had been physically touching were collected. Field collected samples were placed in ziplock bags and stored at approximately 4°C in a cooler with ice packs for transportation back to the lab. Samples were sonicated in 200 ml of sterile water to disrupt biofilms associated with plastic leaf and tomato leaf surfaces. The resulting “wash” water was centrifuged and DNA was extracted from the pellet. No specific permissions were required for collection from these research fields other than the consent of the Wye Research and Education Center (WREC) scientists and extension agents who direct the activities of the WREC Station. The field studies did not involve endangered or protected species.

### 16S rRNA Gene Tailed-End Amplicon Sequencing

16S rRNA gene amplicon sequencing was performed on all samples according to Illumina’s “*Overview of tailed amplicon sequencing approach with MiSeq*” protocol. This two-step PCR approach utilizes sequence specific primers and Nextera DNA Index Kit (Illumina, San Diego, CA) with 16S rRNA gene primers for theV4 region (515f, 805r), GTGCCAGCMGCCGCGGTAA (forward) GGACTACHVGGGTWTCTAAT (reverse)[39].

## Bioinformatic Methods

### 16S amplicon sequence analysis

Raw fastq files reflecting forward reads output by the MiSeq platform were initially filtered for quality and length (≥200bp) using QIIME [40, 41]and spurious hits to the PhiX control genome were identified using BLASTN and removed. Passing sequences were trimmed of the forward primer, and evaluated for chimeras with UCHIME (*de novo* mode) [42], and subsequently filtered for host-related contaminant including chloroplast DNA using the RDP Bayesian classifier [43]. Next a large-scale BLASTN search of the GreenGenes database (v13_05) was performed to identify unknown contaminant sequences. Sequences without a database match of at least 70% identity along 60% of their length were removed. Identified contaminants included a substantial number of mitochondrial DNA. This resulted in an average of 160,000 raw reads per independent replicate with an average length of 221 bases. A full table of sequences throughout stages of quality screening is available in S1 Table.

The final dataset of high-quality 16S rRNA gene amplicon sequences were characterized for diversity and taxonomic composition using QIIME with the GreenGenes database. Sequences were clustered into operational taxonomic units (OTUs) using UCLUST (*de novo*) [44] with a 97% identity threshold. Representative sequences of each cluster were assigned to a taxonomic lineage by the RDP classifier (trained on the GreenGenes 16S database, v13_05) using a minimum threshold of 0.50. Representatives were input to PYNAST [45] to generate a multiple sequence alignment, which was subsequently used to construct a neighbor-joining phylogenetic tree with FastTree [46]. After full characterization of the clean sequence dataset, sampling depth was normalized by rarefaction to 2,500 sequences per sample to include as many independent replicates as possible. The rarefied 16S sequence set was further evaluated by the Resphera Insight protocol to obtain high-resolution taxonomic assignments (Baltimore, MD; www.respherabio.com).

### Statistical analysis

Beta-diversity distance metrics (Bray-Curtis) were computed from rarefied OTU tables and visualized using principal coordinate analysis in QIIME[40]. Hierarchical clustering and visualization were performed in R (v.2.12.0). The false discovery rate (FDR) was employed to control for false positives in comparative statistical testing[34]. nMDS ordination plots were created by applying nonmetric multidimensional scaling to the Bray-Curtis Dissimilarity matrix for the samples. nMDS ordination was achieved by the metaMDS wrapper function from the vegan package (http://CRAN.R-project.org/package=vegan), which uses the monoMDS function from the same package. The ordination was applied such that the data was scaled down to two dimensions. In addition (with a random seed of 246), 20 starting point iterations were performed within the metaMDS function call, leading to a minimum stress level of 0.1575973. Once the ordination was applied, the data was graphed using the ggplot2 package in R. The ellipses seen in the plots are crated using the stat_ellipse function from the ggplot2 package, which assumes a multivariate t distribution[47]. The ellipse is the upper 95^th^ percentile limit of the assumed distribution (95% confidence ellipse).

To test the global null hypothesis of independence for covariate variables (store, phyllo, enviro, and timepoints: T0, T1, T2, T3) and associated response variables (MDS1 and 2), the ctree() function in the party package of R was used to model a conditional inference regression tree onto the data after nMDS scaling. If the null hypothesis cannot be rejected, the process ends. If the null hypothesis is rejected, the covariate with the strongest association (determined using Bonferroni corrected p-values from permutation tests) is selected. A binary split is subsequently performed on the selected covariate. These steps are repeated until all possible nodes are generated using a p value set at 0.1 as a cutoff [31]. An analysis of multivariate homogeneity of group dispersions was also performed using the Vegan R package (betadisper) to provide permutation-based tests of dispersion homogeneity. Additionally, the Bray-Curtis distance matrix was used with ADONIS in R to perform a two-factor PERMANOVA analysis evaluating both time point and environment type (Control/Phyllo). ADONIS identified both time and type as significantly associated with total community composition (P < 0.001), with time influencing composition more so than type.

To evaluate differences in microbial networks among control and phyllosphere communities, we computed Spearman’s correlation coefficients for all defined genera and their corresponding statistical significance, corrected using FDR[34]. Those correlations with significant P-values (P < 0.05) were included for comparative analysis and visualized using Cytoscape v3.x (www.cytoscape.org).

### Data Submission

All 16S rRNA gene fastq files have been deposited in the SRA of NCBI associated with accession number [SRP043640]. All metadata has been submitted according to MIMARKS (minimum information about a marker gene sequence)[48].

## Supporting Information

S1 Fig Bacterial Families in Control and Phyllosphere. Most abundant bacterial families identified using 16S rRNA gene amplicons for all independent replicates of control (C) and phyllosphere (P) at all time-points. Taxonomy was assigned using the RDP classifier trained on the GreenGenes database.
(TIF)

S2 Fig Multivariate Homogeneity of Group Dispersions. Permutation-based tests of dispersion homogeneity were performed using the Vegan R package (betadisper). Significant differences in dispersion were identified between T0 compared to T1 and T1 compared to T2 (P<0.007 for each comparison; *permutest* in Vegan package). No significant differences in dispersions among time-points within each environment were identified (most likely due to limited group sizes).
(TIF)

S1 Table Raw Sequence Data and Preprocessing Details. Full report of pre-processing and quality screening of sequences used for downstream analyses.
(XLSX)

## References

[1] Jackson CR, Denney WC. Annual and seasonal variation in the phyllosphere bacterial community associated with leaves of the southern magnolia (Magnolia grandiflora). Microbial Ecology. 2011;61(1):113–22. 10.1007/s00248-010-9742-2 20809288

[2] Jackson EF, Echlin HL, Jackson CR. Changes in the phyllosphere community of the resurrection fern, Polypodium polypodioides, associated with rainfall and wetting. FEMS Microbiology Ecology. 2006;58(2):236–46. Epub 2006/10/27. FEM152 [pii] 10.1111/j.1574-6941.2006.00152.x . 17064265

[3] Jager ES, Wehner FC, Korsten L. Microbial ecology of the mango phylloplane. Microbial Ecology. 2001;42(2):201–7. Epub 2002/05/25. 10.1007/s002480000106 . 12024283

[4] Kadivar H, Stapleton AE. Ultraviolet radiation alters maize phyllopshere bacterial diversity. Microbial Ecology. 2003;43:353–61. 10.1007/s00248-002-1065-5 12704563

[5] Lambais MR, Crowley DE, Cury JC, Bull RC, Rodrigues RR. Bacterial diversity in tree canopies of the Atlantic forest. Science. 2006;312(5782):1917 Epub 2006/07/01. 312/5782/1917 [pii] 10.1126/science.1124696 . 16809531

[6] Morris CEK, L L.. Fifty years of phyllosphere microbiology: significant contributions to research in related fields In: Lindow SE, editor. Phyllosphere Microbiology. St. Louis, MO: APS Press; 2004.

[7] Ottesen AR, Gonzalez Pena A, White JR, Pettengill JB, Li C, Allard S, et al Baseline survey of the anatomical microbial ecology of an important food plant: Solanum lycopersicum (tomato). BMC Microbiology. 2013;13:114 Epub 2013/05/28. 10.1186/1471-2180-13-114 23705801 PMC3680157

[8] Ottesen AR, White JR, Skaltsas DN, Newell MJ, Walsh CS. Impact of organic and conventional management on the phyllosphere microbial ecology of an apple crop. Journal of Food Protection. 2009;72(11):2321–5. . 19903395 10.4315/0362-028x-72.11.2321

[9] Redford AJ, Bowers RM, Knight R, Linhart Y, Fierer N. The ecology of the phyllosphere: geographic and phylogenetic variability in the distribution of bacteria on tree leaves. Environmental Microbiology. 2010. 10.1111/j.1462-2920.2010.02258.x PMC3156554 20545741

[10] Reisberg EE, Hildebrandt U, Riederer M, Hentschel U. Phyllosphere bacterial communities of trichome-bearing and trichomeless Arabidopsis thaliana leaves. Antonie van Leeuwenhoek. 2012;101(3):551–60. 10.1007/s10482-011-9669-8 22080429

[11] Smit E, Leeflang P, Glandorf B, Dirk van Elsas J, Wernars K. Analysis of fungal diversity in the wheat rhizosphere by sequencing of cloned PCR-amplified genes encoding 18S rRNA and temperature gradient gel electrophoresis. Applied and Environmental Microbiology. 1999;65(6):2614 10347051 10.1128/aem.65.6.2614-2621.1999 PMC91386

[12] Vorholt JA. Microbial life in the phyllosphere. Nature Reviews Microbiology. 2012;10(12):828–40. 10.1038/nrmicro2910 23154261

[13] Yang CH, Crowley DE, Borneman J, Keen NT. Microbial phyllosphere populations are more complex than previously realized. P Natl Acad Sci USA. 2001;98(7):3889–94. . 10.1073/pnas.051633898 PMC31148 11274410

[14] Brandl MT. Fitness of human enteric pathogens on plants and implications for food safety. Annual Review of Phytopathology. 2006;44(1):367–92. 10.1146/annurev.phyto.44.070505.143359 . 16704355

[15] Telias A, White J, Pahl D, Ottesen A, Walsh C. Bacterial community diversity and variation in spray water sources and the tomato fruit surface. BMC Microbiology. 2011;11(1):81. 21510867 10.1186/1471-2180-11-81 PMC3108269

[16] Ottesen A, Telias A, White JR, Newell MJ, Pahl D, Brown EW, et al. Bacteria of tomatoes managed with well water and pond water: Impact of agricultural water sources on carposphere microbiota.

[17] Moulas C, Petsoulas C, Rousidou K, Perruchon C, Karas P, Karpouzas DG. Effects of systemic pesticides imidacloprid and metalaxyl on the phyllosphere of pepper plants. BioMed Research International. 2013;2013. 10.1155/2013/969750 PMC3690639 23841101

[18] Ottesen AR, Gorham S, Pettengill JB, Rideout S, Evans P, Brown E. The impact of systemic and copper pesticide applications on the phyllosphere microflora of tomatoes. Journal of the Science of Food and Agriculture. 2015;95(5):1116–25. 10.1002/jsfa.7010 25410588 PMC4368374

[19] Yashiro E, McManus PS. Effect of streptomycin treatment on bacterial community structure in the apple phyllosphere. PloS one. 2012;7(5):e37131 10.1371/journal.pone.0037131 22629357 PMC3357425

[20] Marine SC, Pagadala S, Wang F, Pahl DM, Melendez MV, Kline WL, et al The growing season, but not the farming system, is a food safety risk determinant for leafy greens in the mid-Atlantic region of the United States. Applied and Environmental Microbiology. 2015;81(7):2395–407. 10.1128/AEM.00051-15 25616798 PMC4357962

[21] Pagadala S, Marine SC, Micallef SA, Wang F, Pahl DM, Melendez MV, et al Assessment of region, farming system, irrigation source and sampling time as food safety risk factors for tomatoes. International Journal of Food Microbiology. 2015;196:98–108. 10.1016/j.ijfoodmicro.2014.12.005 25540859

[22] Perazzolli M, Antonielli L, Storari M, Puopolo G, Pancher M, Giovannini O, et al Resilience of the natural phyllosphere microbiota of the grapevine to chemical and biological pesticides. Applied and Environmental Microbiology. 2014;80(12):3585–96. 10.1128/aem.00415-14 24682305 PMC4054146

[23] Williams TR, Moyne A-L, Harris LJ, Marco ML. Season, irrigation, leaf age, and inoculation influence the bacterial diversity in the lettuce phyllosphere. PloS one. 2013;8(7):e68642 10.1371/journal.pone.0068642 23844230 PMC3699665

[24] Osono T, Mori A. Seasonal and leaf age-dependent changes in occurence of phyllosphere fungi of giant dogwood. Mycoscience. 2005;(46).

[25] Thompson IPBMJF, J.S.; Fermor T.R.; Lilley A.K.; Lynch J.M.; McCormack P.J.; McQuilken M.P.; Purdy K.J.; Rainey P.B.; Whipps J.M. Quantitative and qualitative seasonal changes in the microbial community from the phyllosphere of sugar beet (Beta vulgaris). Plant and Soil. 1993;(150):177–91.

[26] Copeland JK, Yuan L, Layeghifard M, Wang PW, Guttman DS. Seasonal community succession of the phyllosphere microbiome. Molecular Plant Microbe Interactions. 2015;28(3):274–85. 10.1094/MPMI-10-14-0331-FI 25679538

[27] Kembel SW, Mueller RC. Plant traits and taxonomy drive host associations in tropical phyllosphere fungal communities. Botany. 2014;92 10.1139/cjb-2013-0194

[28] Knief C, Ramette A, Frances L, Alonso-Blanco C, Vorholt JA. Site and plant species are important determinants of the Methylobacterium community composition in the plant phyllosphere. Isme J. 2010;4 10.1038/ismej.2010.9 20164863

[29] Laforest-Lapointe I, Messier C, Kembel SW. Host species identity, site and time drive temperate tree phyllosphere bacterial community structure. Microbiome. 2016;4(1):1–10. 10.1186/s40168-016-0174-1 27316353 PMC4912770

[30] Maignien L, DeForce EA, Chafee ME, Eren AM, Simmons SL. Ecological succession and stochastic variation in the assembly of Arabidopsis thaliana phyllosphere communities. mBio. 2014;5(1):e00682–13. 10.1128/mBio.00682-13 24449749 PMC3903271

[31] Hothorn T, Hornik K, Zeileis A. Unbiased recursive partitioning: A conditional inference framework. Journal of Computational and Graphical Statistics. 2006;15(3):651–74.

[32] Innerebner G, Knief C, Vorholt JA. Protection of Arabidopsis thaliana against Leaf-Pathogenic Pseudomonas syringae by Sphingomonas Strains in a Controlled Model System. Applied and Environmental Microbiology. 2011;77(10):3202–10. 10.1128/aem.00133-11 21421777 PMC3126462

[33] Rivas R, Abril A, Trujillo ME, Velázquez E. Sphingomonas phyllosphaerae sp. nov., from the phyllosphere of Acacia caven in Argentina. International journal of systematic and evolutionary microbiology. 2004;54(6):2147–50. 10.1099/ijs.0.63102-0 15545449

[34] Benjamini Y, Hochberg Y. Controlling the false discovery rate—a practical and powerful approach to multiple testing. J Roy Stat Soc B Met. 1995;57(1):289–300. .

[35] Santl Temkiv T, Karlson UG, Lever M, Finster K. The in situ study of active bacterial cells and their sources during atmospheric dispersal. ISME Meeting Abstracts. 2014.

[36] Galès A, Latrille E, Wéry N, Steyer J-P, Godon J-J. Needles of pinus halepensis as biomonitors of bioaerosol emissions. PloS one. 2014;9(11):e112182 10.1371/journal.pone.0112182. PMC4224445. 25379901 PMC4224445

[37] Vokou D, Vareli K, Zarali E, Karamanoli K, Constantinidou HI, Monokrousos N, et al Exploring biodiversity in the bacterial community of the Mediterranean phyllosphere and its relationship with airborne bacteria. Microbial Ecology. 2012;64(3):714–24. 10.1007/s00248-012-0053-7 . 22544345

[38] Williams TR, Marco ML. Phyllosphere microbiota composition and microbial community transplantation on lettuce plants grown indoors. mBio. 2014;5(4). 10.1128/mBio.01564-14 25118240 PMC4145687

[39] Caporaso JG, Lauber CL, Walters WA, Berg-Lyons D, Huntley J, Fierer N, et al Ultra-high-throughput microbial community analysis on the Illumina HiSeq and MiSeq platforms. ISME J. 2012;6(8):1621–4. http://www.nature.com/ismej/journal/v6/n8/suppinfo/ismej20128s1.html. 10.1038/ismej.2012.8 22402401 PMC3400413

[40] Caporaso JG, Bittinger K, Bushman FD, DeSantis TZ, Andersen GL, Knight R. PyNAST: a flexible tool for aligning sequences to a template alignment. Bioinformatics. 2010;26(2):266–7. Epub 2009/11/17. 10.1093/bioinformatics/btp636 19914921 PMC2804299

[41] Kuczynski J, Stombaugh J, Walters WA, González A, Caporaso JG, Knight R. Using QIIME to analyze 16S rRNA gene sequences from microbial communities. Current Protocols in Microbiology. 2012:1E. 5.1-E. 5.20. 10.1002/9780471729259.mc01e05s27 PMC4477843 23184592

[42] Edgar RC, Haas BJ, Clemente JC, Quince C, Knight R. UCHIME improves sensitivity and speed of chimera detection. Bioinformatics. 2011. 10.1093/bioinformatics/btr381 PMC3150044 21700674

[43] Wang Q, Garrity GM, Tiedje JM, Cole JR. Naive Bayesian classifier for rapid assignment of rRNA sequences into the new bacterial taxonomy. Applied and Environmental Microbiology. 2007;73(16):5261 17586664 10.1128/AEM.00062-07 PMC1950982

[44] Edgar RC. Search and clustering orders of magnitude faster than BLAST. Bioinformatics. 2010;26(19):2460–1. Epub 2010/08/17. 10.1093/bioinformatics/btq461 . 20709691

[45] Caporaso JG, Kuczynski J, Stombaugh J, Bittinger K, Bushman FD, Costello EK, et al QIIME allows analysis of high-throughput community sequencing data. Nature Methods. 2010;7(5):335–6. 10.1038/nmeth.f.303 20383131 PMC3156573

[46] Price MN, Dehal PS, Arkin AP. FastTree: computing large minimum evolution trees with profiles instead of a distance matrix. Mol Biol Evol. 2009;26(7):1641–50. Epub 2009/04/21. 10.1093/molbev/msp077 19377059 PMC2693737

[47] Wickham H. ggplot2: elegant graphics for data analysis: Springer Science & Business Media; 2009.

[48] Yilmaz P, Kottmann R, Field D, Knight R, Cole JR, Amaral-Zettler L, et al Minimum information about a marker gene sequence (MIMARKS) and minimum information about any (x) sequence (MIxS) specifications. Nature biotechnology. 2011;29(5):415–20. 10.1038/nbt.1823 21552244 PMC3367316

