# Supplementary figures and images for: Using a Control to Better Understand Phyllosphere Microbiota
Source: PLoS One. 2016 Sep 26;11(9):e0163482. doi: 10.1371/journal.pone.0163482 (PMC5036865; doi:10.1371/journal.pone.0163482)

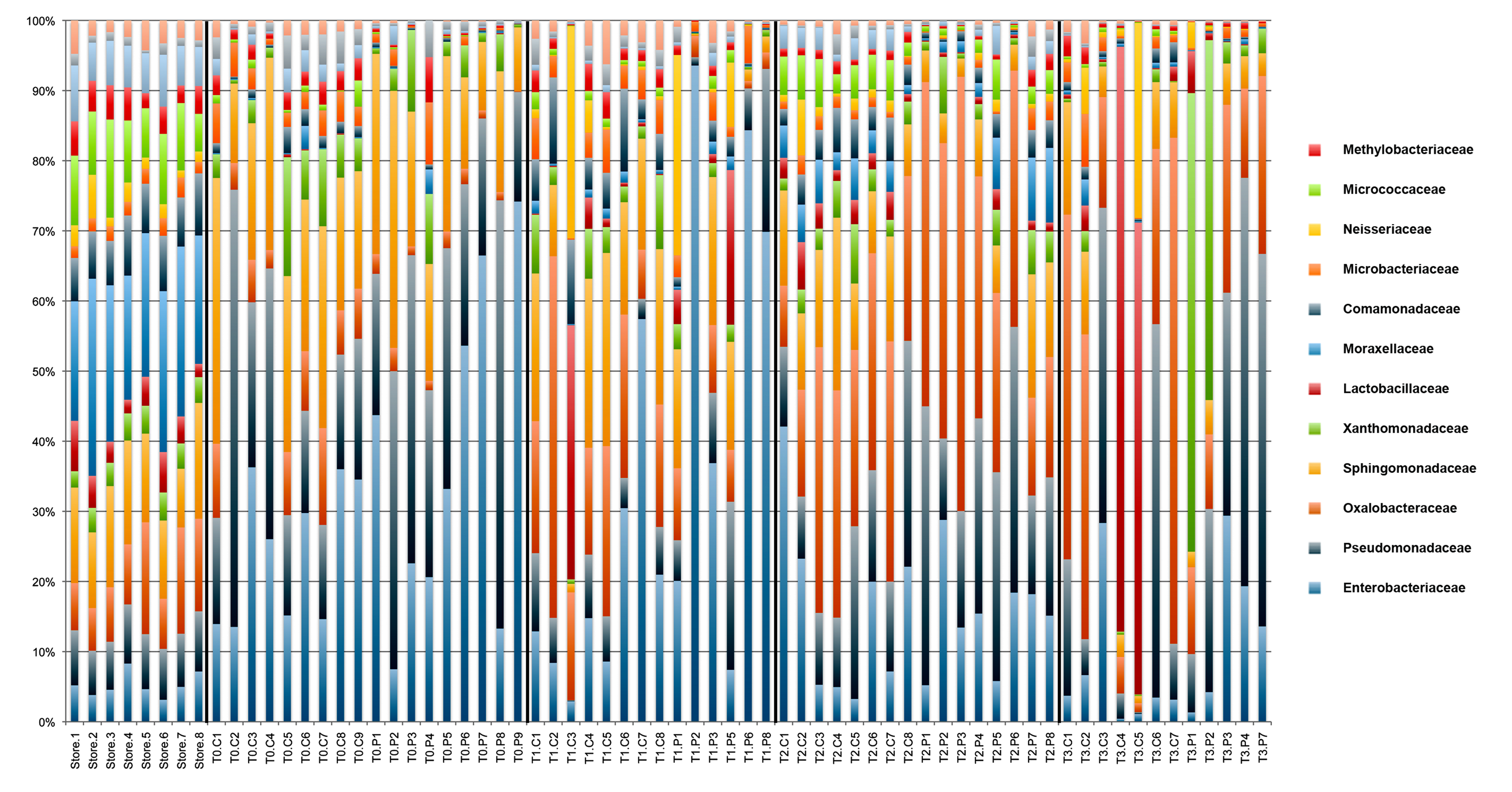

Supplement: S1 Fig — Most abundant bacterial families identified using 16S rRNA gene amplicons for all independent replicates of control (C) and phyllosphere (P) at all time-points. Taxonomy was assigned using the RDP classifier trained on the GreenGenes database. (TIF) [file pone.0163482.s001.tif]

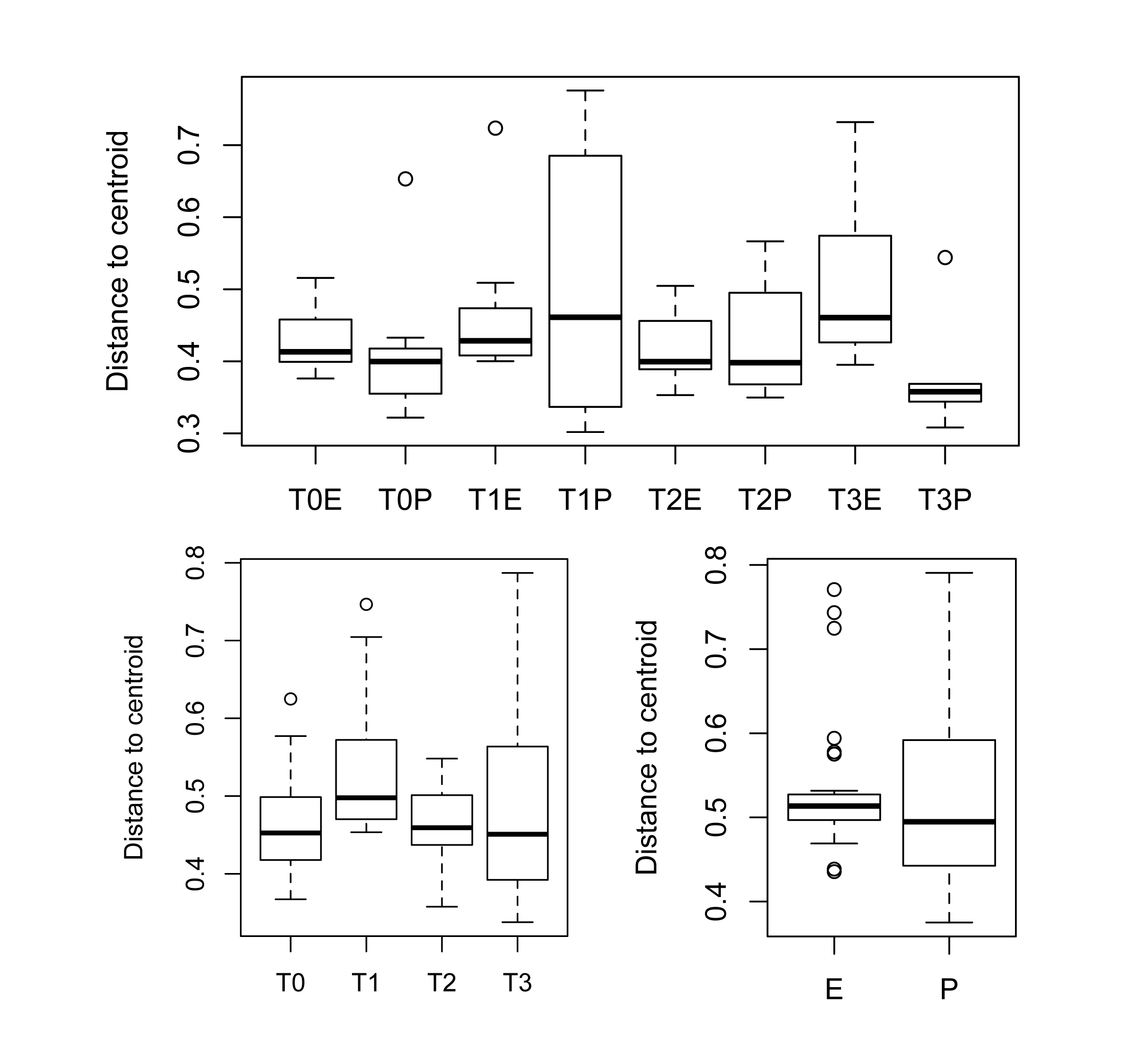

Supplement: S2 Fig — Permutation-based tests of dispersion homogeneity were performed using the Vegan R package (betadisper). Significant differences in dispersion were identified between T0 compared to T1 and T1 compared to T2 (P<0.007 for each comparison; permutest in Vegan package). No significant differences in dispersions among time-points within each environment were identified (most likely due to limited group sizes). (TIF) [file pone.0163482.s002.tif]
